# Maternal lipid levels in pregnant women without complications in developing risk of large for gestational age newborns: a study of meta-analysis

**DOI:** 10.12688/f1000research.26072.2

**Published:** 2021-01-29

**Authors:** Muhammad Pradhiki Mahindra, Mahendra Tri Arif Sampurna, Muhammad Pradhika Mapindra, Apriska Mega Sutowo Putri

**Affiliations:** 1 Faculty of Medicine, Hasanuddin University, Makassar, South Sulawesi, 90245, Indonesia; 2 Department of Pediatrics, Faculty of Medicine, Airlangga University, Surabaya, East Java, 60115, Indonesia; 3 Faculty of Medicine, University of Sebelas Maret, Surakarta, Central Java, 57126, Indonesia

**Keywords:** GDM, healthy women, LGA, maternal lipids, non-LGA

## Abstract

**Background: **Circulating into foetal circulation across the placental barrier, abnormal maternal serum lipids predispose neonates to metabolic dysfunction and thereafter affect the steroid metabolism and functions of extra-embryonic foetal tissues.

**Methods: **A systematic review was conducted by searching PubMed–MEDLINE and the Cochrane library between January 2010 and January 2020. The included studies were English case control studies that described original data on at least one raw lipid measurement during pregnancy in healthy women who delivered large for gestational age (LGA) newborns and in healthy women with non-LGA newborns. The data extracted from 12 studies were pooled, and the weighted mean difference (WMD) in lipid levels was calculated using random effects models. A meta-analysis was performed to identify sources of heterogeneity and to describe the significant value of the collected studies.

**Results: **Of 649 published articles identified, a total of 12 met the inclusion criteria **.** Compared with women who had non-LGA newborns, those who had LGA newborns had significantly higher triglyceride (TG) levels (WMD = 0.28, 95% CI −0.02 to 0.54) and lower high density lipoprotein cholestrol (HDL-C) levels (WMD = 0.08, 95% CI −0.13 to −0.03), but not have significantly lower high-density lipoprotein cholesterol (LDL-C) levels. Moreover, the levels of total cholesterol, low-density lipoprotein cholesterol, and very low density lipoprotein cholesterol (VLDL-C) were inconsistent between both groups.

**Conclusions: **High levels of TG and low levels of HDL-C could cause births of LGA newborns whereas maternal serum of TC, LDL-C and VLDL-C cannot be used as predictor of LGA.

## Introduction

The early stages of pregnancy involve endocrine and metabolic changes and is an important period for placenta formation and foetal development. Epidemiological studies have shown that excessive lipid exposure in the maternal intrauterine environment can affect the development of foetal organs and lead to maternal metabolic impairment ^1^. Abnormalities in maternal serum lipids have been highly correlated with birth weight and may be a cause of neonatal metabolic dysfunction ^2^. The prevalence of foetal macrosomia in developed countries ranges from 5% to 20%, and several studies have reported that gestational diabetes mellitus (GDM) and maternal obesity were strongly associated with the risks for low and high birth weights. Disturbances in maternal metabolism affect blood glucose and other maternal macronutrients, such as lipids, and subsequently affect the development of the foetus ^2– 4^. The role of triglycerides (TG) is yet to be completely understood, but a cohort study in Amsterdam reported that maternal TG concentrations during the early stages of pregnancy were linearly related with the prevalence of large for gestational age (LGA) newborns ^5^. Macrosomic foetuses may develop stillbirth and are at risk for neonatal mortality ^5– 7^. Therefore, LGA newborns have increased risk for developing type 2 diabetes, cardiovascular diseases and hypertension in their adult age ^5– 9^.

High maternal serum lipid levels have been shown to increase the likelihood of pregnancy problems, such as GDM, pre-eclampsia and pre-term delivery ^2^. Moreover, increase in the levels of TG, total cholesterol (TC), high-density lipoprotein cholesterol (HDL-C), low-density lipoprotein cholesterol (LDL-C) and very low-density lipoprotein cholesterol (VLDL-C) can account for adverse outcomes of normal gestation. Maternal serum lipids are transferred through the placenta, suggesting that they can affect foetal sterol metabolism and the metabolic functions of extra-embryonic foetal tissues ^6, 7, 9^. These studies implied how essential lipid levels are to foetal development. The impact of high maternal lipid levels on foetal birth weight remains barely recognised in clinical practice, although this is known to be a cause of cardiovascular disease and diabetes ^2^.

Previous studies have suggested that pregnant women with GDM and normal blood glucose levels had an increased risk for delivering LGA newborns ^10^. The positive association between early maternal hypertriglyceridemia and LGA newborns in low-risk women is well recognised in some studies ^6^. To further investigate the relationship between maternal lipid levels and LGA newborns, we conducted a systematic review of existing cohort studies to determine the potential role of maternal lipid levels as a risk factor for uncomplicated pregnancy related to LGA newborn delivery.

## Methods

### Eligibility criteria

**Figure 1.**
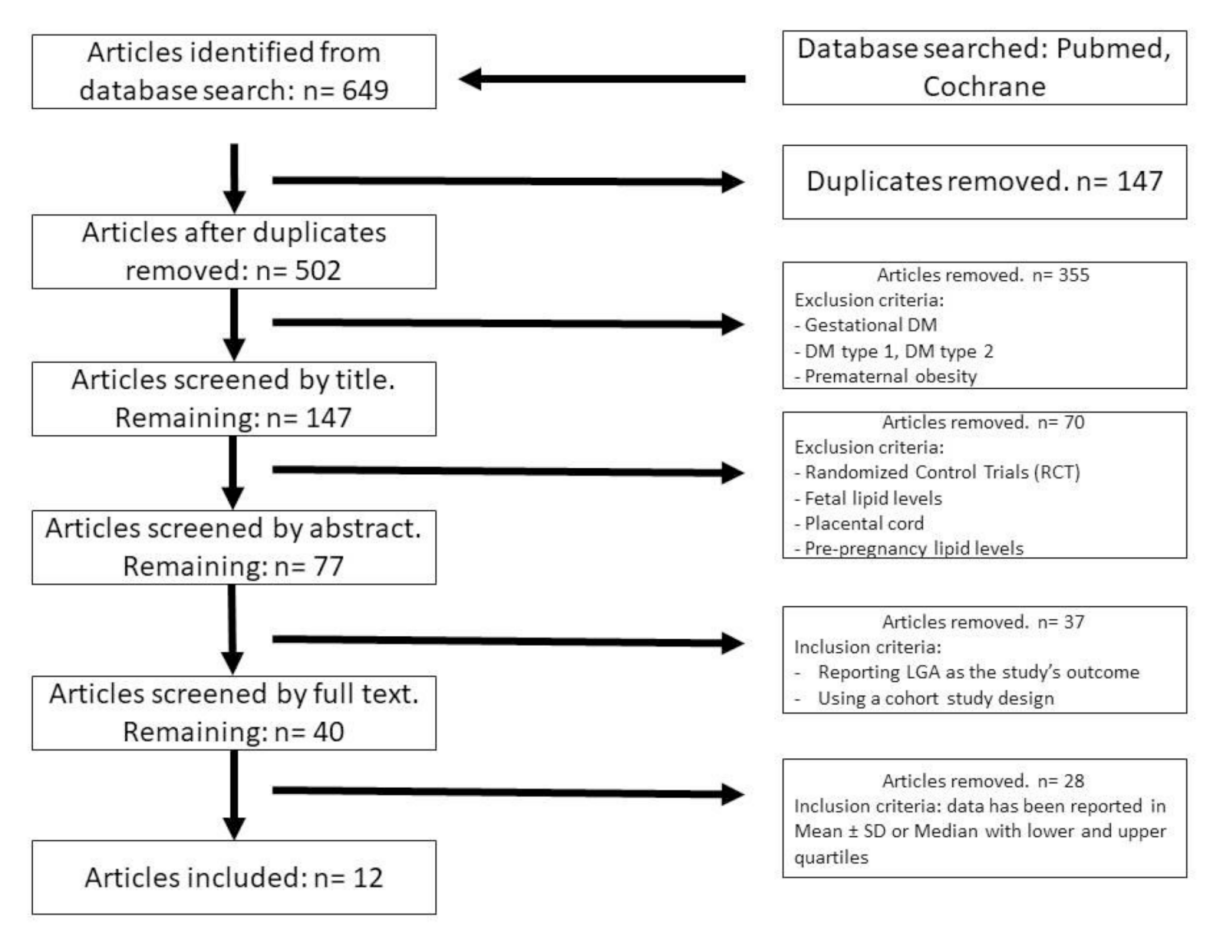
Flowchart of the Literature Review Process.

This study is reported in accordance with the Preferred Reporting Items for Systematic Review and Meta-Analysis Protocols (PRISMA P) guidelines ( Figure 1; *Reporting guidelines* ^11^). Only research papers written in English between January 2010 and January 2020 were included in our search. We retrieved several papers with abstracts that mentioned an association between maternal TG and LGA newborns. For additional citations, references were collected from the included articles. Then, we determined the eligibility of the included studies using a critical appraisal skill programme (CASP) checklist for cohort model to determine the quality of the included studies. Based on the assessment using CASP tools, we finally included 12 articles to assess and review. The variables extracted from the literature are shown in Table 2. Full-text articles were acquired and evaluated for eligibility. 

### Literature search

We conducted our search, which was conducted from April 2020 to June 2020, in the following databases: PubMed (MEDLINE), Library of Michigan University and the Cochrane library. We applied search strings, including combinations of search terms, as keywords placed in the titles or abstracts of the studies ( Table 1). The keywords we used for the search strategy were as follows: 1. maternal lipid profile, lipid profile or lipoprotein and 2. LGA or large for gestational age.

**Table 1.** Electronic search strings.

Data Base	Keywords (Search Strategy)
PubMed	(Lipid profile[all fields] OR Triglycerides[MeSH Terms] OR Cholesterol[MeSH] AND LGA[all fields] OR Large for Gestational Age[all fields]
Michigan Library	(Maternal Lipid profile[all fields] OR Triglycerides[MeSH Terms] OR Cholesterol[MeSH] AND Large for Gestational Age[all fields]
Cochrane Library	(Lipid profile[all fields] OR Triglycerides[all fields] OR Cholesterol[MeSH] AND LGA[all fields] OR Large for Gestational Age[all fields]

### Study selection

We included studies that assessed the relationship between maternal serum TG levels during early to late pregnancy and LGA newborn delivery by healthy women or women who had no confounding factors, such as obesity, GDM, type 1 diabetes mellitus (T1DM), type 2 diabetes mellitus (T2DM), intake of medications that can alter lipid levels and maternal obesity. Lipid profiles, including TG, TC, HDL-C, LDL-C and VLDL-C, were measured from healthy pregnant women who had the outcome LGA newborn delivery. We did not include unpublished studies, letter to the editor, commentary, supplementary materials and conference paper. We excluded studies that did not show any relevance or similarity with our study purposes. This study evaluated maternal serum TG levels measured during pregnancy but not pre-conception TG levels.

### Data extraction and analysis

Maternal serum lipid levels in mmol/L and mg/dL were compared between LGA and non-LGA newborns. The study design, study population, method of data collection, gestational age at sampling and serum lipid levels were evaluated for all the selected studies. We measure lipid levels in mmol/l to make them homogenous so all lipid level measurements being reported in mg/dl were converted to mmol/L ^12^. The reported mean and standard deviation (SD) values in the selected studies were recorded. However, we found several studies that reported their findings in the form of median and interquartile range; we used a digital calculator to change these into estimated mean and SD ^13^.

### Data abstraction

All authors recorded and reviewed the collected articles. The main authors determined the study design, time frame and criteria for the included studies. The other authors helped retrieve the articles and process the data with statistical analyses. We decided to include studies that had prospective and retrospective cohort designs.

### Statistical methods

First, we measured the maternal lipid levels with the standard unit of mmol/L in all included studies; data on maternal lipid levels in mg/dL were converted to mmol/L. Thereafter, we used the Review Manager version 5.3 (RevMan) software designed by Cochrane for statistical analyses. The mean difference and analysed statistical significances of the reported maternal serum lipid levels were calculated and evaluated in terms of impact on the outcome of LGA delivery. I ^2^ statistics was performed to assess heterogeneity.

## Results

### Study characteristics and assessment of risk bias

Our search retrieved 649 articles, 147 of which were independently identified as duplications and thus leaving 502 articles. Subsequently, we decided to select 77 of 502 articles that had titles and abstracts that were related to the measurement of lipoprotein levels in pregnant women and its impact on neonates. Of the 77 articles, 40 articles that had suitable research methods and outcomes were read in full text. We found that 28 of the 40 articles did not indicate mean ± SD or median with upper and lower quartiles for maternal lipid level measurements. Thus, leaving 12 articles that were suitable for inclusion. We cross-checked the remaining articles to ensure that original studies were reported. Detailed information on author’s name, publication year, sample size, study design, the determined lipid level and the definitions of LGA was recorded and tabulated in Microsoft Excel 2010 software ( Table 2 and Table 3).

**Table 2.** Characteristics of the included studies.

Study	Study design	Year of publication	Total number of samples (n)	LGA (n)	Non- LGA (n)	Gestational age at sampling (weeks)	LGA definition	Biochemical lipid (s) determined
Vrijkotte ^9^	Prospective Cohort	2012	4,008	96	3,912	Singleton pregnancies, first trimester	Neonatal birth weight above 90th percentile for gestational age, regardless of sex groups, from the Dutch Perinatal Registration.	Non-fasting TC and TG
Mitra ^14^	Prospective Cohort	2012	50	10	40	Singleton pregnancies, third trimester	Neonatal birth weight above 90th percentile for gestational age-corrected birth weight curve.	TC, TG, HDL, LDL,
Parlakgumus ^15^	Prospective Cohort	2013	411	26	385	Singleton pregnancies, second trimester	Neonatal birth weight above 90th percentile for gestational age on Lubchenco growth charts.	Fasting TG, VLDL, HDL
Harville ^16^	Prospective Cohort	2014	325	35	290	Singleton pregnancies	Neonatal birth weight in the top 10% for gestational age in the study population.	Fasting TG
Lin Hou ^17^	Prospective Cohort	2014	2,790	554	2,236	Singleton pregnancies, third trimester	Neonatal birth weight above 90th percentile for gestational age based on birth weight percentiles by sex and gestational age in Southern China	Fasting TC, HDL-C, LDL-C, TG
Kui Ye ^18^	Prospective Cohort	2015	1204	331	873	Singleton pregnancies, third trimester	Neonatal birth weight above 90th percentile for gestational age	Fasting TC, TG, HDL- C, LDL-C
Wang ^3^	Retrospective Cohort	2017	5,218	856	4,362	Singleton pregnancies, first trimester	Neonatal birth weight above 90th percentile for gestational age based on birth weight percentiles by sex and gestational age in Southern China	Non fasting TC, TG, HDL-C, LDL-C
Farias ^6^	Prospective Cohort	2017	188	36	152	Singleton pregnancies,	Neonatal birth weight above 90th percentile of the INTERGROWTH-21st curves.	Fasting HDL-C, LDL-C,
Geraghty ^7^	Prospective Cohort	2017	327	96	231	singleton pregnancies	Neonatal birth weight above 90th percentile of the INTERGROWTH-21st curves.	, TG
Pazhohan ^19^	Prospective Cohort	2017	951	248	703	Singleton pregnancies, first trimester	Neonatal birth weight above 90th percentile of the INTERGROWTH-21st curves.	Fasting TC, HDL-C, LDL-C, TG
Liang ^4^	Prospective Cohort	2018	2,839	2,405	435	Singleton pregnancies	Neonatal birth weight above 90th percentile of the INTERGROWTH-21st curves.	Fasting TG,
Lee ^20^	Prospective Cohort	2019	623	68	555	Singleton pregnancies, first trimester	Neonatal birth weight >90th percentile for gestational age using data derived from Korean population.	Fasting TC, TG, HDL- C, LDL-C

**Table 3.** Mean values of lipid profiles during pregnancy in women with LGA newborns and controls.

Study	LGA vs. Non-LGA	Triglyceride (mmol/L)	Total cholesterol (mmol/L)	HDL-C (mmol/L)	LDL-C (mmol/L)	VLDL (mmol/L)
Vrijkotte ^9^	LGA	1.44 ± 0.61	5.06 ± 0.91	-	-	
	Non-LGA	1.32 ± 0.54	4.98 ± 0.86	-	-	
Mitra ^14^ ^a^	LGA	2.66± 0.88	1.27 ± 0.22	1.7 ± 0.24	2.85 ± 0.25	1.23± 0.43
	Non-LGA	2.14± 0.63	1.61 ± 0.42	1.77 ± 0.25	2.95 ± 0.25	1.02 ± 0.31
Parlakgumus ^15^^*^^a^	LGA	1.61± 0.92	3.82 ± 0.85	1.27 ± 0.23	2.2 ± 0.88	0.73 ± 0.41
	Non-LGA	1.59± 1.16	4.66 ± 1.83	1.61 ± 0.42	2.85 ± 1.68	0.75 ± 0.50
Harville ^16^	LGA	1.2 ± 0.7	-	-	-	-
	Non-LGA	1.1 ± 0.5	-	-	-	-
Hou ^17^	LGA	2.15± 0.52	6.22 ± 0.49	1.70±0.24	2.96 ± 0.44	-
	Non-LGA	1.23± 0.06	6.33 ± 0.47	1.77±0.25	3.09 ± 0.42	-
Wang ^3^	LGA	1.26 ± 0.68	4.54 ± 0.80	1.71 ± 0.48	2.36 ± 0.68	-
	Non-LGA	1.10 ± 0.69	4.46 ± 0.80	1.73 ± 0.45	2.30 ± 0.66	-
Farias ^6^ ^a^	LGA	4.87 ± 1.91	3.58 ± 2.00	1.27 ± 0.19	2.53 ± 0.41	-
	Non-LGA	4.32 ± 2.30	3.51 ± 0.2	1.23 ± 0.21	2.49 ± 0.56	-
Geraghty ^7^	LGA	1.86 ± 0.14	-	-	-	-
	Non-LGA	1.66 ± 0.16	-	-	-	-
Pazhohan ^18^ ^a^	LGA	2.27± 1.18	5.23 ± 0.8	-	-	-
	Non-LGA	1.82± 0.48	5.08 ± 0.77	-	-	-
Liang ^4^	LGA	2.15 ± 0.52	-	-	-	-
	Non-LGA	1.23 ± 0.06	-	-	-	-
Lee ^20^ ^*a^	LGA	1.38± 0.19	4.42 ± 0.29	1.62 ± 0.17	2.15 ± 0.20	-
	Non-LGA	1.26 ± 0.17	4.43 ± 0.26	1.68 ± 0.13	2.17 ± 0.21	-
Kui Ye ^18^	LGA	3.1 ± 1.2	6.6 ± 1.3	2.30 ± 0.5	3.40 ± 0.80	-
	Non-LGA	2.90 ± 1.2	6.6 ± 1.4	2.40 ± 0.5	3.30 ± 0.80	-

*The mean was calculated by inputting the median, lowest range and highest range to the estimating calculator based on SP Hozo ^13^. ^a^The data was converted from mg/dL to mmol/L using a standard measuring unit ^12^.

As shown in Table 2, the baseline characteristics of the included studies were explained. There were 12 prospective cohort studies that reported a total of 17,731 cases, 4,430 of which included LGA newborns. Maternal lipid profiles were measured in the first trimester in four articles ^3, 9, 19, 20^; in the second trimester in one article ^15^; in the last trimester in three articles ^14, 17, 18^ and in any of the gestational weeks in the remaining articles ^4, 6, 7, 16^.

Most of the studies ^4, 6, 7, 19^ used the INTERGROWTH-21st definition of >90th percentile for LGA newborns. Some studies in China ^3, 17^ used a referred percentile standard based on a Chinese population, and some ^14, 16, 20^ used their country’s definition of LGA. A detailed list of the implemented eligibility criteria for each study is shown in Table 2, and the mean values of the determined biochemical lipids are presented in Table 3. There were 12 included studies that investigated the effects of lipid profile in pregnant women who had no complications on LGA newborn delivery. The exposures of maternal lipid profile included TG (N = 12), TC (N = 9), HDL-C (N = 7), LDL-C (N = 7) and VLDL-C (N = 2). Based on the analysis of the RevMan tool, we found that the investigated studies that analysed maternal TG, TC, HDL-C and LDL-C had an I ^2^ of more than 50%; for this reason, we used a random effects model. On the other hand, we used a fixed effect model to assess the studies that investigated maternal VLDL-C, because the I ^2^ was below 50%.

### Pregnancy triglyceride samples

There were 11 studies that assessed TG levels during pregnancy; 4,761 case subjects and 14,174 control subjects were included. Maternal serum TG levels in the first trimester were found to be significantly associated with LGA infants, according to three of the included studies ^3, 19, 20^. 

One study reported that maternal serum TG levels in the second trimester were significantly related with the risk for LGA newborns before and after data adjustment ^9^. On the other hand, another study on a similar population ^15^ showed a non-significant correlation between maternal serum TG levels and the risk for LGA newborns. Furthermore, two studies ^7, 17^ reported an association between maternal serum TG levels and LGA occurrence. The remaining studies measured TG levels in the first, second and third trimesters and reported significant associations between maternal serum TG levels and the risk for LGA newborns ^4, 7, 16^.

**Figure 2.**
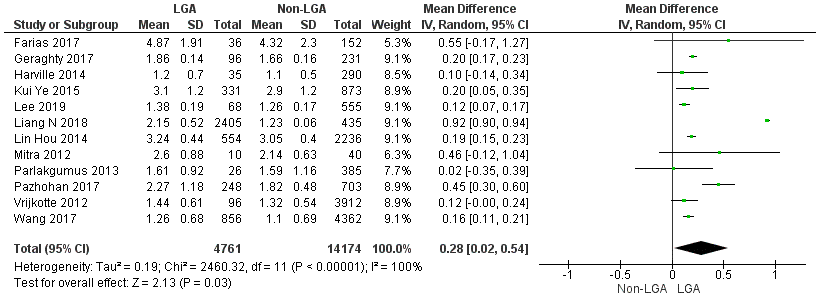
Forest Plot of Maternal TG level exposure and LGA outcome.

Figure 2 shows the comparison of the mean differences of the included studies. Random effects model meta-analysis showed that the pooled weighted mean difference was 0.28 mmol/L (95% CI −0.02 to 0.54), and significant heterogeneity was observed (Tau² = 0.19; Chi²= 2460.32, I² = 100%, p = 0.03).

### Total cholesterol

Data on 2,225 patients and 13,218 controls from nine studies ^3, 6, 9, 14, 15, 17– 20^ were included to evaluate the relationship between TC and LGA newborns. In contrast to all the studies that reported an insignificant association between TC levels and LGA, Wang reported that abnormal levels of maternal TC in the first trimester were significantly associated with the event of LGA infants ^3^. In fact, some reports were insufficient to prove a significant correlation between TC level and the risk for LGA newborns, whereas other reports found non-significant associations between TC levels and the risk for LGA newborns in the first, second and third trimesters ^6, 17^.

**Figure 3.**
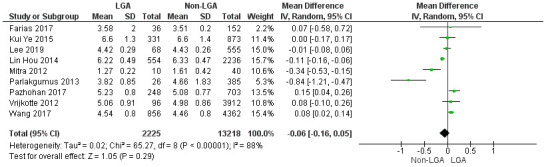
Forest Plot of Maternal TC exposure and LGA outcome.

Based on the random effects model meta-analysis ( Figure 3 and Table 4), the included studies had a pooled weighted mean difference of −0.06 mmol/L (95% CI −0.16 to 0.05) and heterogeneity (Tau ^2^ = 0.02, Chi² = 65.27, I² = 88%) with p value = 0.26.

**Table 4.** Mean differences in TG, TC, HDL-C, LDL-C and outcome among the LGA newborns.

Study	Triglyceride		Total Cholesterol		High-Density Lipoprotein Cholesterol		Low-Density Lipoprotein Cholesterol		Very Low-Density Lipoprotein Cholesterol	
	Weight %	Mean Difference IV, Random, 95% CI	Weight %	Mean Difference IV, Random, 95% CI	Weight %	Mean Difference IV, Random, 95% CI	Weight %	Mean Difference IV, Random, 95% CI	Weight %	Mean Difference IV, Random, 95% CI
Farias ^6^	5.3	0.55 [-0.17, 1.27]	2.2	0.07 [-0.58, 0.72]	14.2	0.04 [-0.03, -0.11]	12.0	0.04 [-0.12, 0.20]	-	Not estimable
Geraghty ^7^	9.1	0.20 [0.17, 0.23]			-	Not estimable	-	Not estimable	-	Not estimable
Harville ^16^	8.5	0.10 [-0.14, 0.34]	-	Not estimable	-	Not estimable	-	Not estimable	-	Not estimable
Kui Ye ^18^	8.9	0.20 [0.05, 0.35]	11.4	0.00 [-0.17, 0.17]	14.9	-0.10 [-0.16, -0.04]	15.7	0.10 [-0.00, 0.20]	-	Not estimable
Lee ^20^	9.1	0.12 0.07, -0.17]	15.0	-0.01 [-0.08, 0.06]	16.9	-0.06 [-0.10, 0.02]	18.6	--0.02 [-0.07, 0.03]	-	Not estimable
Liang N ^4^	9.1	0.92 [0.90, 0.94]	-	Not estimable	-	Not estimable	-	Not estimable	-	Not estimable
Lin Hou ^17^	9.1	0.19 [0.15, 0.23]	15.7	-0.11 [-0.16, -0.06]	18.3	-0.07 [-0.09, -0.05]	19.0	-0.12 [-0.16, -0.08]	-	Not estimable
Mitra ^14^	6.3	0.46 -0.12, 1.04]	10.6	-0.34 [-0.53, -0.15]	6.6	-0.07 [-0.24, -0.10]	11.2	0.00 [-0.17, 0.17]	25.4	0.21 [-0.07. 0.49]
Parlakgumus ^15^	7.7	0.02 [-0.35, 0.39]	5.3	-0.84 [-1.21, -0.47]	11.6	-0.34 [-0.44, -0.24]	4.8	-0.65 [-1.00, -0.30]	74.6	-0.02 [-0.19, 0.15]
Pazohan ^19^	8.9	0.45 [0.30, 0.60]	13.5	0.15 [0.04, 0.26]	-	Not estimable	-	Not estimable	-	Not estimable
Vrijkotte ^9^	8.9	0.12 [-0.00, 0.24]	10.8	0.08 [-0.10, 0.26]	-	Not estimable	-	Not estimable	-	Not estimable
Wang ^3^	10.0	0.16 [0.11, 0.21]	15.4	0.08 [0.02, 0.14]	17.5	-0.02 [-0.05, 0.01]	18.6	0.06 [0.01, 0.11]	-	Not estimable
Total (95% CI)	100.0	0.28 [0.02, 0.54]	100.0	-0.06 [-0.16, 0.05]	100.0	-0.08 [-0.13, -0.03]	100.0	-0.03 [-0.11, 0.06]	-	Not estimable

### High density lipoprotein - cholesterol

The analysis of HDL-C and risk for LGA newborns included 1,881 patients and 8,603 controls from seven studies ^3, 6, 14, 15, 17, 18, 20^. HDL-C levels in the third trimester of pregnancy were significantly associated with both LGA and SGA infants ^14, 18^. In addition, one study found a significant association in the second trimester ^6^. On the other hand, one study showed HDL-C as the only lipid that was not significantly related with the birth of LGA infants ^3^.

**Figure 4.**
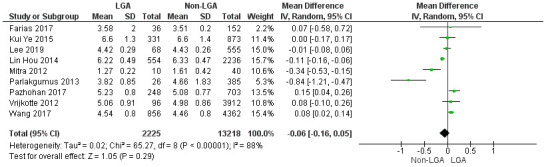
Forest Plot of Maternal HDL-C exposure and LGA outcome.

Figure 4 and Table 4 show the results of the meta-analysis of the included studies. The pooled weighted mean difference was 0.08 (95% CI −0.13 to −0.03), and heterogeneity was found (Tau ^2 ^= 0.00, Chi² = 46.53, I² = 87%), with p = 0.003.

### Low density lipoprotein - cholesterol

Six of the included studies ^3, 6, 14, 15, 17, 18, 20^, which recruited 1,881 patients and 8,603 controls, majority reported no significant correlations between LDL-C concentration and LGA newborns as a neonatal outcome. On the other hand, four studies ^3, 6, 15, 20^ showed that LDL-C concentration was associated with LGA newborns. This association was found during the second and third trimesters of pregnancy ^6^. Furthermore, the study by Wang supported this association by showing that LDL-C concentrations played a significant role in the risk for LGA newborns and that three lipids (TG, TC and LDL-C) were significant contributing factors ^3^.

**Figure 5.**
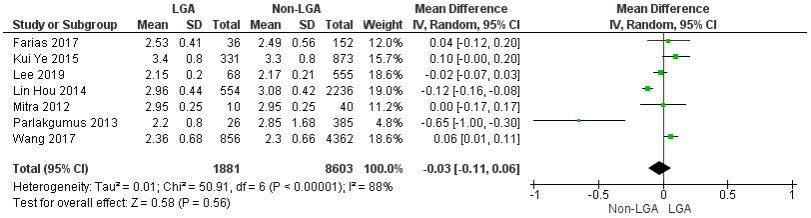
Forest Plot of Maternal LDL-C exposure and LGA outcome.

Figure 5 and Table 4 show that the pooled weight mean difference was −0.03 (95% CI −0.11 to 0.06) and that heterogeneity was found (Tau ^2 ^= 0.01, Chi² = 50.91, I² = 88%), with p = 0.56.

### Very low density lipoprotein - cholesterol

**Figure 6.**
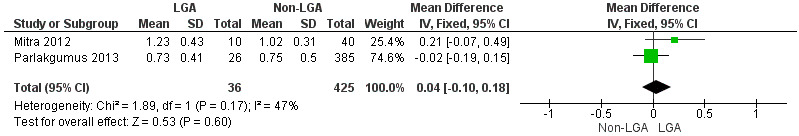
Forest Plot of Maternal VLDL exposure and LGA outcome.

Studies and available information on the impact of VLDL on LGA remain unclear. Nevertheless, two studies that included a total of 36 patients and 425 controls reported that there was no correlation between VLDL and LGA newborns ^14, 15^. Based on our meta-analysis, the level of maternal serum VLDL was not significantly associated with births of LGA newborns (p = 0.60) ( Figure 6).

## Discussion

Data from 12 published articles were evaluated in this systematic review to determine the relationship between lipid values measured during pregnancy and the risk for LGA newborns. Our review presented some valuable findings. We discovered that TC levels were inconsistent in both groups of women who delivered LGA and non-LGA newborns. This finding suggested that TC level as a determinant of LGA newborn delivery is clinically not useful. In support of this result, almost all studies reported that TC levels were similar across the groups. In addition, Parlakgumus ^15^ reported that TC levels in the second trimester took a decisive role on the risk for LGA newborns, compared with the results of many studies.

Many of the studies reported increase in TG levels in women who delivered LGA newborns. Our meta-analysis concluded that maternal TG levels were significantly elevated in women who would deliver LGA neonates. Moreover, maternal HDL-C levels were lower in women with LGA newborns than in those with non-LGA newborns. Therefore, a low level of maternal HDL-C concentration was significantly associated with the risk for LGA newborns. Levels of maternal LDL-C had no significant weight on women who had LGA newborns. Therefore, LDL-C and VLDL-C levels were not significant causative factors of LGA outcomes in pregnant women who had no comorbidities.

Exclusion of all confounding factors, such as T1DM, T2DM, GDM, maternal obesity and excessive gestational weight gain (GWG), which can affect the increased risk for LGA newborns in women who had abnormal lipid profiles, was important. A large prospective study on more than 700 women showed significant correlations of T1DM and HbA1c ≥ 42 mmol/L (6%) during 26 and 34 weeks age of gestation with increased risks of LGA newborns ^21^. Similarly, a retrospective cohort study by Lisa *et al.* showed considerably higher rates of LGA newborns in women with T1DM (39%) than in women with T2DM (17%); their multivariate analysis on non-Caucasian women demonstrated an increased risk for LGA newborns in women who had T1DM (OR = 4.07; 95% CI 1.46 to 11.35) and T2DM (OR = 2.47; 95% CI 1.15 to 5.32) ^22^.

A reported study on 175 women with T1DM in the United States discovered similar rates of LGA newborn delivery in women who had HbA1C of >6.5% and <6.5%, suggesting the likelihood of T1DM as a contributing factor ^23^. A cohort study on multi-ethnic groups revealed that GDM and relatively high pregnancy BMI were linked with an increased risk for LGA newborn delivery. The prevalence of LGA newborns among women with GDM was highest in African, American and Hispanic women and lowest in Asian, Filipino and White women ^24^. In another study on GDM, women who had elevated fasting plasma glucose levels were at a relatively high risk for having LGA newborns ^25^. An analysis of 23,000 women in the Hyperglycaemia and Adverse Pregnancy Outcomes study discovered that the macrosomia prevalence in non-obese women was 6.7% in 1,244 patients without GDM and 10.2% in 2,791 patients with GDM. The investigators found that the frequency of macrosomia was 50% higher in women with GDM than in women without GDM in both the non-obese and obese groups ^26^. Moreover, abnormal pre-pregnancy BMI significantly increased the risk for LGA neonates. A previous longitudinal study reported that compared the groups of women with T1DM and T2DM and healthy women found a positive association between maternal serum TG and LGA infants regardless of glycemic levels condition ^27^. Fasting maternal hypertriglyceridemia could be used as a significant predictor of LGA infants that is independent of maternal BMI, weight gain, and blood glucose levels ^28^. 

Our review could not exclude women with excessive GWG, because this was not reported in the majority of the included studies. Therefore, we assumed that excessive GWG may have affected our results. Some studies showed that excessive GWG in pregnant women who had no complications increased the risk for delivering LGA newborn; compared with women who had uncomplicated pregnancies, those who exceeded the GWG recommendation had three and six times higher risk for macrosomia births ^29^. The expected association of pre-pregnancy BMI and GWG with maternal and foetal outcomes showed that GWG of >16 kg led to an increased risk for delivering LGA neonates ^30^. A study by Lu *et al.* reported that high second trimester GWG was significantly related with a relatively high risk for LGA newborns ^31^. The probability of giving birth to an LGA newborn increased by 6.9% per kilogram of maternal weight gain, and the odds ratio was 1.249 for GWG beyond the recommended amount ^32^. Similarly, another study found that the odds ratio for delivering LGA newborns was higher for non-diabetic Caucasian women with BMIs <25 or >25 than in women with GDM and normal BMIs ^33^.

Lower TG and Higher HDL-C levels are linked to the physical inactivity, a tendency to less responsive to regular exercise. A program of exercise training is reported effective to alter the concentrations of lipoprotein, which therefore prompt the lipoprotein levels to be in the expected range ^34, 35^. As one of the most simple blood measurements, lipid levels especially LGA and HDL-C could be used as a routine blood test during the pregnancy for fetal programming. Normalization of lipid levels should be one of the main targets during pregnancy. Physical activity and dietary adjustment such as habitual fish consumption would be an effective approach to reduce maternal TG levels and increase HDL-C levels ^36, 37^

Moreover, maternal lipid profiles are not only informative to predict neonatal outcomes, but also tends to be important information that is integral to pregnant women’s metabolic status, including act as a potential predictor for GDM in pregnant women. High concentrations of TG, TC, and LDL were found in women diagnosed with GDM throughout the second trimester ^38^. Another prospective cohort had also demonstrated that women who were exhibiting GDM in the second trimester, had shown higher levels of TG, TC, and LDL, and lower levels of HDL during the first trimester, even with normal glycemia and glycated hemoglobin ^39^. These findings emphasized that the role of lipid metabolism is crucial to contribute to the pathogenesis of such metabolic disorders. 

### Strengths and limitations

This review was the first to directly address the association between maternal lipid profiles and the risk for LGA newborns, without any confounding factors. However, it had several weaknesses. First, this review depended on the design and quality of the included studies, regardless of the baseline lipid level, which was crucial to the results of our meta-analysis. Second, our meta-analysis did not distinguish pregnant women based on trimester of pregnancy but described the effects of physiologic changes in lipid metabolism on pregnant women and the risk for LGA newborns throughout the entire pregnancy; it did not consider the confounding factors that may occur in different trimesters. We assumed that our review results might not be sufficient to meet our expectations. Therefore, all of these reasons became researcher biases, which may have resulted in our findings.

### Future studies

Most of the existing observational studies cannot be used to predict the definitive value of the independent contribution of lipid levels to maternal and neonatal outcomes because of the unmeasured confounding factors and methodological limitations. We recommend that future studies analyse women separately based on their non-modifiable characteristics, such as maternal age, race and inherited disorders. Furthermore, we noticed that these observational studies did not exclude women who had excessive GWG, which can contribute to the risk for LGA newborns. Future observational studies must include details on maternal lifestyles and environment to minimise population bias.

## Conclusions

In conclusion, this review demonstrated notable findings from studies on the associations between maternal lipid levels and risk for LGA newborns. Our meta-analysis emphasised that high levels of TG and low levels of HDL-C may affect foetal development and cause births of LGA newborns. On the other hand, maternal serum of TC, LDL-C and VLDL-C cannot be used as predictor of LGA without the other risk factors, such as excessive GWG and insulin resistance. However, we need a better understanding of the relative contributions of other confounding factors, such as gestational age at sampling, maternal age and excessive GWG. We acknowledge that we used exclusion criteria, such as T1DM, T2DM, obesity and hypertension. Excessive GWG was not an exclusion criterion because of the limited amount of studies that excluded such population of women, although we were aware that it may contribute to LGA newborn delivery in healthy women.

## Data availability

### Underlying data

Figshare: Underlying Data - Maternal Lipid Levels on Pregnant Women without Complication in Developing Risk of Large for Gestational Age Newborn: Meta-Analysis study, https://doi.org/10.6084/m9.figshare.13011941.v2 ^40^.

### Reporting guidelines

Figshare: PRISMA checklist for ‘Maternal Lipid Levels on Pregnant Women without Complication in Developing Risk of Large for Gestational Age Newborn: Meta-Analysis study’, https://doi.org/10.6084/m9.figshare.13011803.v2 ^11^.

Data are available under the terms of the Creative Commons Attribution 4.0 International license (CC-BY 4.0).
