# Supplementary material for: Maternal lipid levels in pregnant women without complications in developing risk of large for gestational age newborns: a study of meta-analysis
Source: F1000Res. 2021 Jan 29;9:1213. Originally published 2020 Oct 8. [Version 2] doi: 10.12688/f1000research.26072.2 (PMC7883316; doi:10.12688/f1000research.26072.2)
Supplement: Peer review history

# Peer review: Reviewer response for version 2

**DOI:** 10.5256/f1000research.35486.r78460

**Published:** 2021-02-12

**Authors:** Kian Djien Liem

**Affiliations:** 1 Department of Neonatology, Radboud University Medical Center, Nijmegen, The Netherlands

The authors have made adequate changes in the manuscript as suggested. The mistakes have been corrected. They have made a recommendation for the clinical practise concerning the lipid level of pregnant women.

Are the rationale for, and objectives of, the Systematic Review clearly stated?

Yes

Is the statistical analysis and its interpretation appropriate?

Yes

Are sufficient details of the methods and analysis provided to allow replication by others?

Yes

Are the conclusions drawn adequately supported by the results presented in the review?

Partly

Reviewer Expertise:

neonatal medicine

I confirm that I have read this submission and believe that I have an appropriate level of expertise to confirm that it is of an acceptable scientific standard.

# Peer review: Reviewer response for version 1

**DOI:** 10.5256/f1000research.28773.r72781

**Published:** 2020-12-21

**Authors:** Victor Samuel Rajadurai

**Affiliations:** 1 Department of Neonatology, KK Women's and Children's Hospital, Singapore, Singapore

The authors have done a systematic review of the influence of lipid levels in pregnant women and their effect on the developing fetus particularly, large for gestational age (LGA). The review has clearly addressed the rationale for the review, objectives, characteristics of the participants and outcomes. Sufficient details of the search criteria, methods, inclusion & exclusion criteria and details of the data collection have been clearly defined. Standard statistical methods, applications and interpretations have been used. The conclusions are valid and supported by the results.

 Suggested the following changes:

1. Low levels of HDL Cholesterol may reflect the life style, lack of exercise and relative inactivity of the women included in the articles. Sufficient information regarding this has not been mentioned in the papers included. (To discuss in detail in a separate paragraph).
2. Under “strengths and Limitations”: I would suggest to delete, the third mentioned limitation as this has already been discussed under inclusion criteria.
3. It would be good to discuss whether increased TG levels in the absence of overt GDM or established DM (Type 1 & 2) could be a marker of pre-diabetics in some women who give birth to LGA babies.

Are the rationale for, and objectives of, the Systematic Review clearly stated?

Yes

Is the statistical analysis and its interpretation appropriate?

Yes

Are sufficient details of the methods and analysis provided to allow replication by others?

Yes

Are the conclusions drawn adequately supported by the results presented in the review?

Yes

Reviewer Expertise:

NA

I confirm that I have read this submission and believe that I have an appropriate level of expertise to confirm that it is of an acceptable scientific standard.

# Response

**Published:** 2020-12-23

**Authors:** Mahendra Tri Arif Sampurna

**Affiliations:** Faculty of Medicine Universitas Airlangga, Indonesia

1. Thank you for your suggestion.

 We would like to add this information on discussion section.

 “Lower TG and Higher HDL-C levels are linked to the physical inactivity, a tendency to less responsive to regular exercise. A program of exercise training is reported effective to alter the concentrations of lipoprotein, which therefore prompt the lipoprotein levels to be in the expected range (1,2).

 2. Thank you very much for your corrections. We would like to delete the statement as you suggested.

 3. Thank you very much for your suggestion, we have added the informative statement as you suggested in the discussion section.

 “Maternal lipid profiles are reported as the potential predictor for GDM in pregnant women. High concentrations of TG, TC, and LDL were found in women diagnosed with GDM throughout the second trimester (3).  Another prospective cohort had also demonstrated that women who were exhibiting GDM in the second trimester, had shown higher levels of TG, TC, and LDL, and lower levels of HDL during the first trimester, even with normal glycemia and glycated hemoglobin (4). These findings emphasized that the role of lipid metabolism is crucial to contribute to the pathogenesis of such metabolic disorders.”

 References:

 (1)   (1) C, Després JP, Lamarche B, Bergeron J, Gagnon J, Leon AS, Rao DC, Skinner JS, Wilmore JH, Bouchard C. Effects of endurance exercise training on plasma HDL cholesterol levels depend on levels of triglycerides: evidence from men of the Health, Risk Factors, Exercise Training and Genetics PLEASE NOTE

 If you are an AUTHOR of this article, please check that you signed in with the account associated with this article otherwise we cannot automatically identify your role as an author and your comment will be labelled as a “User Comment”.

 If you are a REVIEWER of this article, please check that you have signed in with the account associated with this article and then go to your account to submit your report, please do not post your review here.

 If you do not have access to your original account, please contact us.

User comments must be in English, comprehensible and relevant to the article under discussion. We reserve the right to remove any comments that we consider to be inappropriate, offensive or otherwise in breach of the User Comment Terms and Conditions. Commenters must not use a comment for personal attacks. When criticisms of the article are based on unpublished data, the data should be made available.

(HERITAGE) Family Study. Arteriosclerosis, thrombosis, and vascular biology. 2001 Jul;21(7):1226-32.

(2) Larrydurstine J, Haskell WL. Effects of exercise training on plasma lipids and lipoproteins. Exercise and sport sciences reviews. 1994 Jan 1;22(1):477-522.

References :

(3)  Correa PJ, Venegas P, Palmeiro Y, Albers D, Rice G, Roa J, Cortez J, Monckeberg M, Schepeler M, Osorio E, Illanes SE. First trimester prediction of gestational diabetes mellitus using plasma biomarkers: a case-control study. Journal of perinatal medicine. 2019 Feb 25;47(2):161-8.

(4)  Li G, Kong L, Zhang L, Fan L, Su Y, Rose JC, Zhang W. Early pregnancy maternal lipid profiles and the risk of gestational diabetes mellitus stratified for body mass index. Reproductive Sciences. 2015 Jun;22(6):712-7.

# Response

**Published:** 2020-12-23

**Authors:** Mahendra Tri Arif Sampurna

**Affiliations:** Faculty of Medicine Universitas Airlangga, Indonesia

Thank you for your correcting our article.We would like to answer the questions.

**Results Section**

 1. We would like to apologize because of several mistakes about the article screening process in Figure 1. We have edited these mistakes and replaced the Figure 1, attached on the revised file

 "Our search retrieved 649 articles, 147 of which wereindependently identified as duplications and thus leaving 502 articles. Subsequently, we decided to select 77 of 502 articles that had titles and abstracts

 that were related to the measurement of lipoprotein levels in pregnant women and its impact on neonates. Of the 77 articles, 40 articles that had suitable research methods and outcomes

 were read in full text".

 .

 2. Thank you for your correction. We changed it into: 

 “ We found that 28 of the 40 articles did not indicate mean ± SD or median with upper and lower quartiles for maternal lipid level measurements. Thus, leaving 12 articles that were suitable for inclusion.

 3. Thank you for your correction. We changed it into

 “ Maternal serum TG levels in the first trimester were found to be significantly associated with LGA infants, according to three of the included"

 4. Thank you for your comment. Actually, this sentence is a mistake and not informative. We decided to delete it because it did not imply our study results

 5. Thank you for your comment. We changed it into :

 "Wang reported that abnormal levels of maternal TC in the first trimester were significantly associated with the event of LGA infants studies”

 6. Thank you for your comments. Due to mistyping, we changed it according to your suggestion

 " HDL-C levels in the third trimester of pregnancy were significantly associated with both LGA and SGA infants"

**Discussion Section**

 7. Thank you for your comments. We would like to change the confusing sentences :

 "A large prospective study on more than 700 women showed significant

 correlations of T1DM and HbA1c ≥ 42 mmol/L (6%) during 26 and 34 weeks

 age of gestation with increased risks of LGA newborns".

 8. As one of the most simple blood measurement, lipid levels especially LGA and HDL-C could be used as a routine blood test during the pregnancy for fetal programming. Normalization of lipid levels should be one of the main targets during the pregnancy. Physical activity and dietary adjustment such as habitual fish consumption would be an effective approach to reduce maternal TG levels and increase HDL-C levels (1,2).

 9. Thank you for your suggestion. We added useful information to support our study and put it in disucssion section.

  "A previous longitudinal study reported that compared the groups of women with T1DM and T2DM and healthy women found a positive association between maternal serum TG and LGA infants regardless of glycemic levels condition (3). Fasting maternal hypertriglyceridemia could be used as a significant predictor of LGA infants that is independent of maternal BMI, weight gain, and blood glucose levels (4).

 References :

 (1) Butler, C. L., Williams, M. A., Sorensen, T. K., Frederick, I. O., & Leisenring, W. M. (2004). Relation between maternal recreational physical activity and plasma lipids in early pregnancy. *American journal of epidemiology*, *160*(4), 350-359.

 (2) Williams, M. A., Frederick, I. O., Qiu, C., Meryman, L. J., King, I. B., Walsh, S. W., & Sorensen, T. K. (2006). Maternal erythrocyte omega-3 and omega-6 fatty acids, and plasma lipid concentrations, are associated with habitual dietary fish consumption in early pregnancy. *Clinical biochemistry*, *39*(11), 1063-1070.

 (3) Göbl, C. S., Handisurya, A., Klein, K., Bozkurt, L., Luger, A., Bancher-Todesca, D., & Kautzky-Willer, A. (2010). Changes in serum lipid levels during pregnancy in type 1 and type 2 diabetic subjects. *Diabetes care*, *33*(9), 2071-2073.

 (4) Kitajima, M., Oka, S., Yasuhi, I., Fukuda, M., Rii, Y., & Ishimaru, T. (2001). Maternal serum triglyceride at 24–32 weeks’ gestation and newborn weight in nondiabetic women with positive diabetic screens. *Obstetrics & Gynecology*, *97*(5), 776-780

# Peer review: Reviewer response for version 1

**DOI:** 10.5256/f1000research.28773.r72787

**Published:** 2020-11-03

**Authors:** Kian Djien Liem

**Affiliations:** 1 Department of Neonatology, Radboud University Medical Center, Nijmegen, The Netherlands

**General comments**

 This is a well-written manuscript. The authors have tried to explore the relationships between maternal lipid levels and the risk of having a large for gestational age (LGA) offspring using a systematic review. The results are of great importance for the obstetricians to be aware of the risk factors for developing LGA newborns. With this knowledge of the risk factors, they can take measures in the pregnant woman to prevent LGA newborns. LGA should be avoided as much as possible, since it is a risk factor for developing metabolic syndrome in adult life.

 The authors have demonstrated that there is an association between maternal lipid levels and risk for LGA newborns, especially high levels TG and low levers HDL-C. But they don’t make any recommendation what we should do for the pregnant women in order to prevent LGA of their offspring’s. Should we determine the lipid level as a routine during pregnancy? But determination of lipid levels during pregnancy could be too late, especially when this is abnormal. Therefore should we give more attention in pre-conception care and recommend normalization of lipid levels, before the women become pregnant? I would like to challenge the authors to make some discussion on the topic about what would be the consequences of their finding.

**Specific comments**

**Introduction:**

 No comments

**Methods:**

 No comments

**Results:**

 Page 4, the data mentioned under Study characteristics and assessment of risk bias don’t match with the data in figure 1. In the text it is mentioned: *“Our search retrieved 649 articles, 147 of which were independently identified as duplications”*.  But in Figure 1 it is mentioned that the number of articles identified from database search is 643 and 183 is identified as duplicate. Which numbers are the right one?

 Thereafter, it is mentioned in the text: *“Subsequently, we decided to select 77 of 147 articles that had titles and abstracts that were related with the measurement of lipoprotein levels in pregnant women and its impact on neonates”.* But this is confusing. Do the authors mean that they select 77 articles from the 147 duplication articles? It doesn’t match with figure 1.

 A few lines further it is mentioned: *“We found that 28 of the 40 articles did not look at the target population, leaving 13 articles that were suitable for inclusion”.* But in figure 1 it is mentioned that 12 articles are included. Which number is the right one?

 In the next paragraph it is mentioned: *“There were 12 prospective cohort studies that reported a total of 17,731 cases, 4,430 of which included LGA newborns”.* It seems that 12 is the right number, like the number in figure 1.

 Page 6 and 7: It is mentioned at the end of page 6: *“Maternal serum TG levels were found to be significantly associated with LGA foetuses in the first trimester, according to three of the included studies.”* . What do the authors mean with LGA foetuses in the first trimester? LGA is defined as birth weight above the 90th percentile for their gestational age and gender. So, it is impossible to speak about LGA fetuses in te first semester. Do they mean the foetuses which in utero grows much larger than average for the  gestational age or fetuses with estimated fetal weight > 90 ^th^ percentile? But this is different than LGA.

 Page 7: It is mentioned: *“Each mmol/L increase in maternal serum TG was found to increase the risk for LGA newborns and reduce the probability of small gestational age newborns”.* Probably is this sentence not complete. How much is the percentage increase of the risk for LGA newborns and percentage decrease of the probability of SGA newborns for each mmol/L increase in maternal serum TG.

 Page 7, under Total cholesterol. It is mentioned: *“Wang reported that abnormal levels of maternal TC were significantly associated with the event of LGA delivery in the first trimester”.* What do the authors mean with the event of LGA delivery in the first trimester. Does it mean miscarriage of a foetus, with a body weight > 90th percentile for the gestational age?

 Page 7 under High density lipoprotein – cholesterol. The authors mention: *“HDL-C levels were reported to be significantly associated with the risk for LGA and SGA foetuses in the third trimester of pregnancy”. *According to the definition, LGA and SGA can only be diagnosed after birth after weighing the newborn's body weight. What do the authors mean with LGA and SGA fetuses in the third trimester? Do they mean the foetuses which estimated fetal weight larger or less than average for the gestational age?

**Discussion**

 Page 8: the authors mention: *“A large prospective study on more than 700 women showed significant correlations of T1DM and HbA1c ≥42 mmol/L (6%) with increased rates of LGA at 26 and 34 weeks age of gestation”.* What do they mean with increased rates of LGA at 26 and 34 weeks age of gestation. Does it mean increased rates of fetuses with estimated fetal weight > 90 ^th^ percentile at 26 and 34 weeks age of gestation?

 Page 8: the authors discuss the relationship between T1DM, T2DM and GDM with LGA newborns. This is a well-known relationship. Therefore, it is the question whether this has any added value to include in the discussion. Perhaps it might be better when the authors will discuss the influence of abnormal maternal lipid level on the body weight of the foetuses of pregnant women with T1DM, T2DM and GDM. Do pregnant women with T1DM, T2DM and GDM and abnormal maternal lipid level have a higher risk for LGA newborns than in pregnant women with T1DM, T2DM and GDM with normal lipid level?

Are the rationale for, and objectives of, the Systematic Review clearly stated?

Yes

Is the statistical analysis and its interpretation appropriate?

Yes

Are sufficient details of the methods and analysis provided to allow replication by others?

Yes

Are the conclusions drawn adequately supported by the results presented in the review?

Partly

Reviewer Expertise:

neonatal medicine

I confirm that I have read this submission and believe that I have an appropriate level of expertise to confirm that it is of an acceptable scientific standard, however I have significant reservations, as outlined above.

# Response

**Published:** 2020-11-23

**Authors:** Mahendra Tri Arif Sampurna

**Affiliations:** Faculty of Medicine Universitas Airlangga, Indonesia

Thank you for your correcting this mistake.

 We would like to answer the questions.

 As one of the most simple blood measurement, lipid levels especially LGA and HDL-C could be used as a routine blood test during the pregnancy for fetal programming. Normalization of lipid levels should be one of the main targets during the pregnancy. Physical activity and dietary adjustment such as habitual fish consumption would be an effective approach to reduce maternal TG levels and increase HDL-C levels (1,2).

 Reference :

 (1) Butler, C. L., Williams, M. A., Sorensen, T. K., Frederick, I. O., & Leisenring, W. M. (2004). Relation between maternal recreational physical activity and plasma lipids in early pregnancy. *American journal of epidemiology*, *160*(4), 350-359.

 (2) Williams, M. A., Frederick, I. O., Qiu, C., Meryman, L. J., King, I. B., Walsh, S. W., & Sorensen, T. K. (2006). Maternal erythrocyte omega-3 and omega-6 fatty acids, and plasma lipid concentrations, are associated with habitual dietary fish consumption in early pregnancy. *Clinical biochemistry*, *39*(11), 1063-1070.

# Response

**Published:** 2020-11-23

**Authors:** Mahendra Tri Arif Sampurna

**Affiliations:** Faculty of Medicine Universitas Airlangga, Indonesia

Thank you for correcting these mistakes in Figure 1

 1. Results section, Page 4, the data mentioned under Study characteristics and assessment of risk bias don’t match with the data in figure 1. In the text it is mentioned: *“Our search retrieved 649 articles, 147 of which* *were independently identified as duplications”*. But in Figure 1 it is mentioned that the number of articles identified from database search is 643 and 183 is identified as duplicate. Which numbers are the right one?

 Thereafter, it is mentioned in the text: *“Subsequently, we decided to select 77 of 147 articles that had*

*titles and abstracts that were related with the measurement of lipoprotein levels in pregnant women*

*and its impact on neonates”. *But this is confusing. Do the authors mean that they select 77 articles

 from the 147 duplication articles? It doesn’t match with figure 1.

 A few lines further it is mentioned: *“We found that 28 of the 40 articles did not look at the target*

*population, leaving 13 articles* *that were suitable for inclusion”. *But in figure 1 it is mentioned that 12

 articles are included. Which number is the right one?

 In the next paragraph it is mentioned: *“There were 12 prospective cohort studies that reported a total*

*of 17,731 cases, 4,430 of which included LGA newborns”. *It seems that 12 is the right number, like the

 number in figure 1.

**Answers :** We would like to apologize because of several mistakes about the article screening process in Figure 1. we would also intend to edit the Figure 1. Regarding the statement, we would revise our statement as follows :

 Our search retrieved 649 articles, 147 of which were independently identified as duplications and thus leaving 502 articles. Subsequently, we decided to select 77 of 502 articles that had titles and abstracts

 that were related to the measurement of lipoprotein levels in pregnant women and its impact on neonates. Of the 77 articles, 40 articles that had suitable research methods and outcomes

 were read in full text. We found that 28 of the 40 articles did not indicate mean ± SD or median with upper and lower quartiles for maternal lipid level measurements. Thus, leaving 12 articles that were suitable for inclusion.

 2. Results section, Page 6 and 7: It is mentioned at the end of page 6: *“Maternal serum TG levels were found to be* *significantly associated with LGA foetuses in the first trimester, according to three of the included* *studies.” *. What do the authors mean with LGA foetuses in the first trimester? LGA is defined as birth weight above the 90th percentile for their gestational age and gender. So, it is impossible to

 speak about LGA fetuses in te first semester. Do they mean the foetuses which in utero grows much larger than average for the gestational age or fetuses with estimated fetal weight > 90th percentile? But this is different than LGA.

**Answers :** Thank you for your correction. We changed the confusing statement to :

 “ Maternal serum TG levels in the first trimester were found to be significantly associated with LGA infants, according to three of the included studies”

 3. Results section, Page 7: It is mentioned: *“Each mmol/L increase in maternal serum TG was found to increase the risk* *for LGA newborns and reduce the probability of small gestational age newborns”. *Probably is this sentence not complete. How much is the percentage increase of the risk for LGA newborns and percentage decrease of the probability of SGA newborns for each mmol/L increase in maternal serum TG.

**Answers : **Thank you very much for your corrections. Actually, this sentence is a mistake and not informative statement. We decided to delete it because it did not imply our study results

 4.  Results section, Page 7 under High density lipoprotein – cholesterol. The authors mention: *“HDL-C levels were* *reported to be significantly associated with the risk for LGA and SGA foetuses in the third trimester of* *pregnancy”. *According to the definition, LGA and SGA can only be diagnosed after birth after weighing the newborn's body weight. What do the authors mean with LGA and SGA fetuses in the

 third trimester? Do they mean the foetuses which estimated fetal weight larger or less than average for the gestational age?

**Answers : **Thank you for your comment and corrections

 We would like to change the confusing statement to :

*Wang reported that abnormal levels of maternal TC in the first trimester were significantly associated with the event of LGA infants*

 5. Results section, Page 7 under High density lipoprotein – cholesterol. We mention: *“HDL-C levels were* *reported to be significantly associated with the risk for LGA and SGA foetuses in the third trimester of* *pregnancy”.*

**Answers : **Thank you for your corrections. We would like to vhange the statement to :

*HDL-C levels in the third trimester of pregnancy were* *significantly associated with both LGA and SGA infants*

 6. Discussion Section, Page 8: the authors mention: *“A large prospective study on more than 700 women showed significant* *correlations of T1DM and HbA1c* *≥42 mmol/L (6%) with increased rates of LGA at 26 and 34 weeks* *age of gestation*”. What do they mean with increased rates of LGA at 26 and 34 weeks age of gestation. Does it mean increased rates of fetuses with estimated fetal weight > 90th percentile at 26 and 34 weeks age of gestation?

**Answers : **Thank you for the corrections. We would like to apologize for confusing statement. We would like to changed it to :

*A large prospective study on more than 700 women showed significant* *correlations of T1DM and HbA1c* *≥ 42 mmol/L (6%) during 26 and 34 weeks* *age of gestation with increased risks of LGA newborns.*

 7. Discussion section, Page 8: the authors discuss the relationship between T1DM, T2DM and GDM with LGA newborns.

 This is a well-known relationship. Therefore, it is the question whether this has any added value to

 include in the discussion. Perhaps it might be better when the authors will discuss the influence of

 abnormal maternal lipid level on the body weight of the foetuses of pregnant women with T1DM,

 T2DM and GDM. Do pregnant women with T1DM, T2DM and GDM and abnormal maternal lipid

 level have a higher risk for LGA newborns than in pregnant women with T1DM, T2DM and GDM

 with normal lipid level?

**Answers : **Thank you ver much for your suggestion. We would like to add useful information to support our study :

  A previous longitudinal study reported that compared the groups of women with T1DM and T2DM and healthy women found a positive association between maternal serum TG and LGA infants regardless of glycemic levels condition (1). Fasting maternal hypertriglyceridemia could be used as a significant predictor of LGA infants that is independent of maternal BMI, weight gain, and blood glucose levels (2).

 Reference :

 (1) Göbl, C. S., Handisurya, A., Klein, K., Bozkurt, L., Luger, A., Bancher-Todesca, D., & Kautzky-Willer, A. (2010). Changes in serum lipid levels during pregnancy in type 1 and type 2 diabetic subjects. *Diabetes care*, *33*(9), 2071-2073.

 (2) Kitajima, M., Oka, S., Yasuhi, I., Fukuda, M., Rii, Y., & Ishimaru, T. (2001). Maternal serum triglyceride at 24–32 weeks’ gestation and newborn weight in nondiabetic women with positive diabetic screens. *Obstetrics & Gynecology*, *97*(5), 776-780.
